# Structure-based molecular screening and dynamic simulation of phytocompounds targeting VEGFR-2: a novel therapeutic approach for papillary thyroid carcinoma

**DOI:** 10.3389/fphar.2025.1583329

**Published:** 2025-06-09

**Authors:** Shuai Wang, Lingqian Zhang, Wenjun Zhang, Xiong Zeng, Jie Mei, Weidong Xiao, Lijie Yang

**Affiliations:** ^1^ Department of General Surgery, Xinqiao Hospital, The Army Medical University, Chongqing, China; ^2^ Department of Hematology-Oncology, Chongqing University Cancer Hospital, Chongqing, China

**Keywords:** papillary thyroid carcinoma, VEGFR-2, molecular dynamic simulation, angiogenesis, virtual drug screening

## Abstract

Papillary thyroid carcinoma (PTC) is the most prevalent type of thyroid cancer, with aggressive variants presenting major therapeutic challenges. Vascular endothelial growth factor receptor-2 (VEGFR-2) is a key regulator of tumor angiogenesis and is highly expressed in PTC, making it a promising target for therapeutic intervention. This highlights the potential of VEGFR-2 inhibition as an effective strategy for managing PTC. In this study, we employed virtual drug screening, molecular dynamics simulations, and binding free energy calculations to identify potential VEGFR-2 inhibitors from the African natural product database (AfroDb). Our virtual drug screening identified three lead compounds SA_0090, 17.3.1.7.8 and BMC_0005 with a docking scores of −9.04 kcal/mol, −8.96 kcal/mol, and −8.33 kcal/mol respectively, surpassing the control compound (−8.39 kcal/mol). Molecular dynamics simulation analysis confirmed the dynamic stability, structural compactness, and minimal residual fluctuations of the 17.3.1.7.8 and BMC_0005 compounds-VEGFR2 complexes. The binding free energy calculations further supported the strong interactions, with values recorded as −60.3861 ± 0.39 kcal/mol for the control, −52.2732 ± 0.37 kcal/mol for SA_0090, −52.7797 ± 0.62 kcal/mol for 17.3.1.7.8, and −61.476 ± 0.59 kcal/mol for BMC_0005. Additionally, the selected compounds exhibited highly favorable ADMET properties, including optimal water solubility, efficient gastrointestinal absorption, and a non-hepatotoxic profile, all aligning with Lipinski’s rule of five. In conclusion, these findings highlight 17.3.1.7.8 and BMC_0005 compounds as compelling candidates for VEGFR-2 inhibition, offering a promising therapeutic avenue for papillary thyroid carcinoma, warranting further *in vitro* and *in vivo* validation for potential therapeutic use.

## Introduction

Thyroid carcinoma is the most prevalent endocrine malignancy, with Papillary Thyroid Carcinoma (PTC) being the most common subtype, representing 80%–85% of thyroid cancers in both adults and children (Limaiem et al., 2024). Originating from the follicular cells of the thyroid, PTC is typically well-differentiated and has a low, stable mortality rate. However, certain aggressive variants and advanced stages pose a greater risk, often leading to recurrence, metastasis, and poor outcomes (Gordon et al., 2022) (Mao et al., 2025). Notably, PTC with multifocality and early lymph node metastasis shows a recurrence rate of up to 35%, and the 10-year survival rate for advanced cases drops below 50% (Tuttle et al., 2017). Given PTC’s high incidence and the aggressive behavior of some forms, understanding the risk factors, molecular mechanisms, and metastatic processes is essential, as current treatment approaches remain insufficient. The exact cause of papillary thyroid carcinoma (PTC) is unknown, but risk factors include genetic syndromes (e.g., familial adenomatous polyposis), radiation exposure, and iodine imbalance (Zhang and Xu, 2024). Environmental and hormonal factors, such as endocrine disruptors and prolonged estrogen exposure, also play a role. PTC may be asymptomatic or present with a painless thyroid nodule, voice changes, or difficulty swallowing (Harahap and Jung, 2024).

Metastasis is most frequent in Hurthle cell tumors (33%) and is also common in medullary and anaplastic thyroid cancers. Overall, distant metastasis occurs in about 4% of thyroid tumors at diagnosis, while skin metastasis from papillary thyroid cancer is rare (<1%) (Somoza et al., 2013). Vasculogenesis, angiogenesis, tumorigenesis, and inflammation involve multiple physiological and pathological processes driven by factors like fibroblast growth factor, VEGF, HGF, and interleukin-6 (Ghalehbandi et al., 2023). Increased angiogenesis supports tumor growth by stimulating new capillary formation from existing blood vessels (Modi and Kulkarni, 2020). Tyrosine kinases (TKs), particularly VEGFR-2, are key regulators of this process and are overexpressed in various cancers. VEGFR-2 activation triggers signaling pathways that enhance cell survival, proliferation, and growth (Sana et al., 2020). Previous studies have revealed that VEGFR-2 is highly expressed in both nodular hyperplasia and papillary thyroid carcinoma (PTC). Its expression is particularly pronounced in PTC, where it is accompanied by the co-expression of VEGF and VEGFR-1. This suggests that targeting VEGFR could be a valuable strategy for managing papillary thyroid carcinoma (Gogiashvili et al., 2024).

The VEGFR-2 gene, located at chromosome 4q11-12, encodes a receptor tyrosine kinase composed of 1,356 amino acids. This receptor exists in three forms: a non-glycosylated version (150 kD), an intermediate glycosylated form (200 kD), and a fully mature glycosylated receptor (230 kD). Notably, only the mature glycosylated VEGFR-2 is capable of initiating intracellular signal transduction (Shah et al., 2025). VEGFR2 (Vascular Endothelial Growth Factor Receptor 2) plays a central role in cancer angiogenesis by mediating the effects of VEGF-A. When VEGF-A binds to VEGFR2, it activates several downstream signaling pathways (like PI3K/AKT, MAPK, and Src pathways) that promote endothelial cell survival, proliferation, migration, vascular permeability, and capillary formation. This leads to the development of an abnormal, leaky, and disorganized tumor vasculature, which supports tumor growth and metastasis (Ghalehbandi et al., 2023). VEGFR-2 tyrosine kinase inhibitors completely blocked VEGF-induced angiogenesis and significantly reduced bFGF-induced angiogenesis in both *in vivo* and *in vitro* models. In endothelial cell invasion assays, these inhibitors suppressed VEGF- and bFGF-driven invasion by 100% and about 90%, respectively (Tille et al., 2001; Sarabipour et al., 2016).

VEGFR-2 is overactive in cancer cells but not in normal cells, making it a prime target for selective cancer therapies. Blocking VEGFR-2 activation with inhibitors prevents tumor angiogenesis without affecting healthy tissues. Several FDA-approved VEGFR-2 inhibitors have been developed to treat various cancers by restricting blood vessel growth. However, these drugs often cause significant side effects, leading researchers to explore new small molecules with better efficacy and fewer adverse effects (Claesson‐Welsh and Welsh, 2013). Computational chemistry has become a key tool in drug discovery, aiding in the design, optimization, and ADMET evaluation of potential VEGFR-2 inhibitors (Suleimen et al., 2021) (El-Adl et al., 2021) (Alanazi et al., 2021). Natural products (NPs) represent a vast reservoir of chemically diverse and biologically active molecules, many of which have served as essential drugs or lead compounds for treating various diseases (Shahrajabian et al., 2022) (Mehrbod et al., 2021). For centuries, traditional medicine has relied on these naturally derived compounds, demonstrating their therapeutic potential. Compared to synthetic drugs, natural compounds often exhibit greater selectivity, reduced toxicity, and cost-effectiveness, making them attractive candidates for drug development (Aware et al., 2022). African natural products offer unique chemical diversity and structural novelty, often guided by traditional medicinal knowledge. They show strong potential against infectious and drug-resistant diseases and are underrepresented in global libraries, making them a valuable source for novel drug discovery. In this study, we employed virtual drug screening, molecular dynamics simulations, and binding free energy calculations to identify potential VEGFR-2 inhibitors from the African natural product database. Inhibiting VEGFR-2 could serve as an effective therapeutic strategy for managing papillary thyroid carcinoma by suppressing tumor angiogenesis.

## Methodology

### Retrieval and preparation of crystal structure of VEGFR2

The crystal structure of VEGFR2 bound to a native benzimidazole-urea inhibitor (PDB ID: 2OH4) was retrieved from the Research Collaboratory for Structural Bioinformatics Protein Data Bank (RCSB PDB) (https://www.rcsb.org/) (Suleman et al., 2025). To prepare the structure for further analysis, all water molecules were eliminated using PyMOL. Subsequently, hydrogen atoms were incorporated into the protein, and energy minimization was performed using Chimera to refine the overall structure and resolve any steric clashes (Zhang et al., 2021; Sayaf et al., 2023).

### Virtual screening of natural products libraries against VEGFR2 protein

The AfroDb database (https://zinc12.docking.org/pbcs/afronp), comprising a diverse collection of bioactive natural products derived from African medicinal plants, was retrieved and formatted for compatibility (Ntie-Kang et al., 2013). To ensure drug-likeness and minimize toxicity risks, compounds violating Lipinski’s Rule of Five (R5) were filtered out using the FAF4drug online webserver (Sayaf et al., 2023). Before virtual screening with EasyDock Vina 2.0, ligand structures were converted to the.pdbqt format. Open Babel was employed to assign atomic charges, atom types, and prepare ligands. Receptor preparation involved generating grid maps in AutoGrid, defining the known active site residues (Glu883, Val914, Cys917, Asp1044, and Phe1045) (Elkaeed et al., 2022). Preliminary docking was conducted using the AUTODOCK4 algorithm with an exhaustiveness value of 16 for initial screening, followed by a more refined evaluation at exhaustiveness 64 to eliminate false positives. The top 54 compounds, with docking scores ranging from −7.601 to −8.839 kcal/mol, proceeded to IFD (induced fit docking) via AutoDockFR. This method incorporated receptor flexibility, covalent docking, and using force fields like Amber or CHARMM, along with force-field-based scoring functions. Afterword, the default IFD parameters were applied (Ravindranath et al., 2015). In the rigid docking phase (initial screening), the receptor was kept fixed, and only ligand flexibility was considered. However, In the flexible docking (IFD) stage, key amino acid residues around the binding site were allowed to move (side-chain flexibility), while the rest of the receptor remained relatively rigid. Scoring functions in AutoDockFR were used both before and after the flexible adjustment. Initially, standard AutoDock scoring functions estimated the binding poses. After incorporating receptor flexibility, the force-field-based scoring recalculated the binding energies, providing a refined and more accurate ranking of ligand binding affinities. Finally, the top four candidates were subjected to structural validation and molecular dynamics simulations. PyMOL and Schrödinger Maestro (academic version) were used for visual analysis to further assess binding interactions and stability (Khan et al., 2022).

### Molecular dynamic simulation analysis of VEGFR2-ligands complexes

Molecular dynamic simulation of VEGFR2-ligands complexes was performed by using AMBER21 software known for its sophisticated computational techniques and offering great detail for the stepwise or holistic visualization of biomolecular systems. The tLeap module was used to make coordinates and topology files corresponding to each protein-ligand complex (Case et al., 2005; Salomon‐Ferrer et al., 2013). The system was solvated in a TIP3P water box (14 Å) and counterions (Na + or Cl−) were added for neutralization. To generate the necessary topology and force field modification (frcmod) files for the ligands, the parameters were assigned according to GAFF2 force field by using the antechamber and parmchk2 tools. By alternating between the conjugate gradient and steepest descent approaches, energy minimization was carried out in phases, enabling the system to achieve a stable conformation while minimizing unfavorable steric conflicts. The next phase included increasing the temperature, which was kept at the setpoint using coupling algorithms like Langevin dynamics or Berendsen thermostat. For the production phase, each system underwent a 400-nanosecond simulation with NPT (constant pressure and temperature) or NVT (constant volume and temperature) ensemble. This stage enabled evaluation of the protein-ligand interactions’ dynamics over a period, which was necessary for understanding binding strength and conformational changes throughout the duration of the interactions (Salomon-Ferrer et al., 2013).

### Post-simulation analysis of VEGFR2-ligands complexes

For the post-simulation analysis (residual fluctuation, dynamic stability, compactness, hydrogen bonds) of shortlisted compounds-VEGFR2 complexes, we used the CPPTRAJ and PTRAJ packages (Roe and Cheatham, 2013). At the residue level, we assessed the degree of flexibility using Root Mean Square Fluctuation (RMSF) analysis. Rather than studying the total movement of the complex, the RMSF technique focused on tracking the movement of specific residues throughout a given period of time. The RMSF values were obtained using the following equation:

$$Thermal\;factor\;or\;B-factor=\left[\left(8\pi **2\right)/3\right]\left(msf\right)$$

The structural compactness of the complexes over the simulation period was assessed by computing the radius of gyration (Rg) using the following mathematical formula:

$$R_{gyr}^{2}=\frac{1}{M}\sum_{i=1}^{N}m_{i}\left(r_{i}‐R^{2}\right)$$

$$M=\sum_{i=1}^{N}m_{i}$$

$${R=N}^{-1}\sum_{i=1}^{N}r_{i}$$

Additionally, to determine the stability of the complexes, the Root Mean Square Deviation (RMSD) was calculated. The RMSD values provided insight into the overall structural deviations throughout the simulation, using the mathematical expression below:

$$RMSD=\sqrt{\frac{\sum d^{2}i=1}{N_{atoms}}}$$

### Post-simulation binding free energy calculation of VEGFR2-ligand complexes

To calculate the binding free energies of lead compounds-VEGFR2 complexes we used the MMPBSA. PY script (Sayaf et al., 2023). For the binding free energies calculation, we selected the last 1,000 frames of the MD simulation. Numerous studies use this computational method extensively to assess the Total Binding Energy (TBE) of various ligands (Sayaf et al., 2024) (Khan et al., 2025). The free energy of the ligand in its unbound solvated form (*G*_*ligand, solvated*_), the receptor in its solvated state (*G*_*receptor, solvated*_), and the completely solvated complex (*G*_*complex, solvated*_) were all evaluated. The relationship between these energy components can be further represented through the following equation:

$${∆G}_{bind}=G_{\left(complex,solvated\right)}-G_{\left(ligand,solvated\right)}-G_{\left(VEGFR2,solvated\right)}$$ (1)

To delve deeper into the specific energy contributions, we reformulated Equation as:

$$G=E_{Molecular\;Mechanics}-G_{solvated}-TS$$ (2)

To calculate the specific energy term, the formula was restructured as follows:

$${∆G}_{bind}={∆E}_{Molecular\;Mechanics}+{∆G}_{solvated}-∆TS={∆G}_{vaccum}+{∆G}_{solvated}$$ (3)

$${∆E}_{Molecular\;Mechanics}={∆E}_{\mathrm{int}}+{∆E}_{electrostatic}+{∆E}_{vdW}$$ (4)

$$∆G_{solvated}=∆G_{Generalized\;born}+∆G_{surface\;area}$$ (5)

$$∆G_{surface\;area}=\gamma .SASA+b$$ (6)

### Lipinski’s rule, and pharmacokinetics analysis

Lipinski’s Rule of Five, which specifies important characteristics for oral bioavailability, is crucial in drug design. These include molecular weight ≤500, hydrogen bond donors ≤5, hydrogen bond acceptors ≤10, and log P ≤5 (Pollastri, 2010). In drug discovery, Lipinski’s Rule of Five is a key rule that aids in predicting the oral bioavailability of possible therapeutic candidates. To check adherence to these computational criteria, we used SwissADME (http://www.swissadme.ch/), a web tool that predicts physicochemical parameters, pharmacokinetics, drug-likeness, and drugability in medicinal chemistry (Daina et al., 2017). Moreover, we conducted an ADMET (Absorption, Distribution, Metabolism, Excretion, and Toxicity) analysis using pkCSM (https://biosig.lab.uq.edu.au/pkcsm/) (Pires et al., 2015), which provided insights into critical pharmacokinetic parameters such as skin sensitivity, hepatotoxicity, solubility, blood-brain barrier permeability, and intestinal absorption. Our goal was to find lead phytocompounds with promising drug-like qualities so that they may be developed further in the pharmaceutical industry by using these computational approaches.

## Results and discussion

**FIGURE 1:**
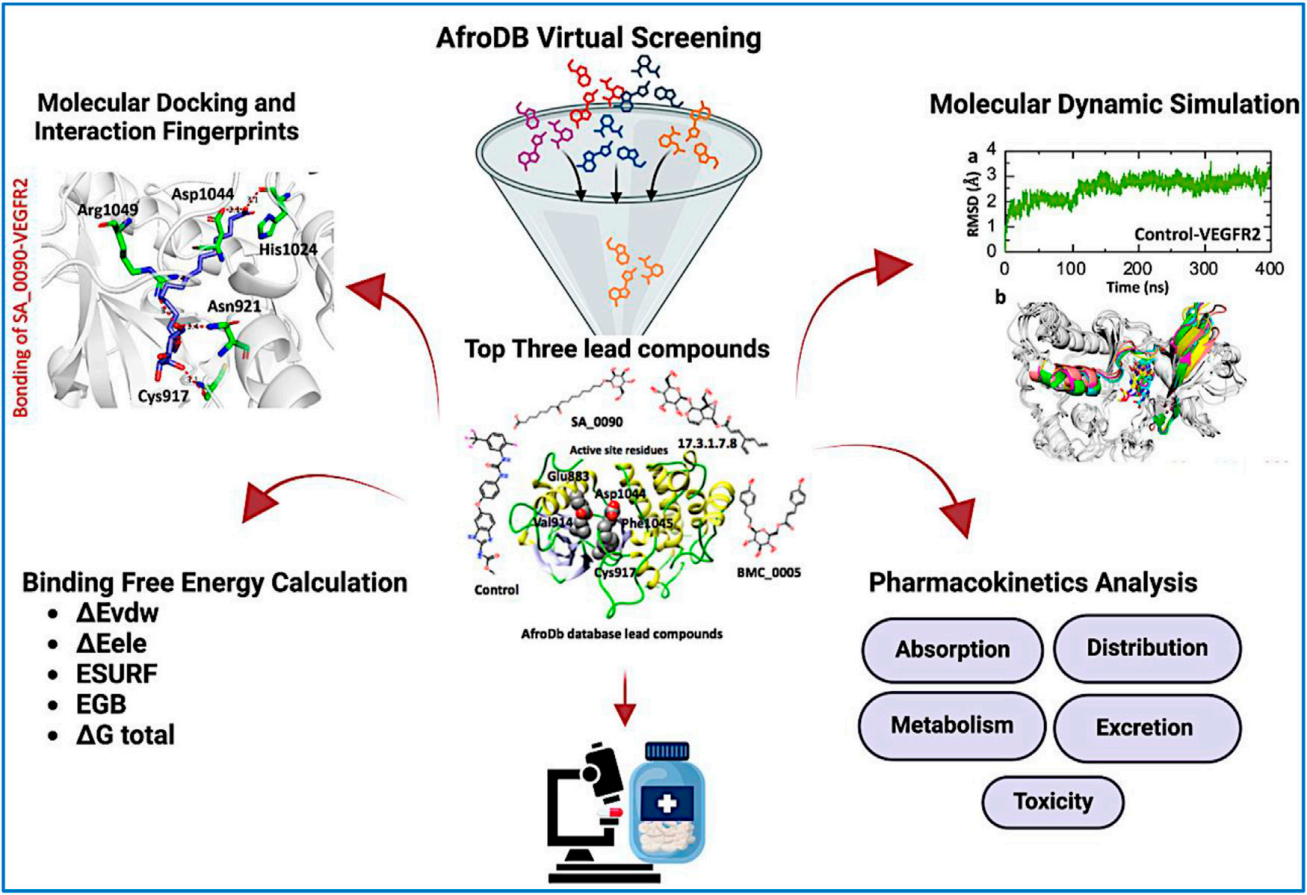
Overall work flow of the study, illustrating the virtual screening and validation of the selected compounds through MD simulation and BFE calculation.

Papillary Thyroid Carcinoma (PTC) is the most common type of thyroid cancer, where aggressive variants often lead to recurrence with a poor prognosis (Zhang and Xu, 2024). The pro-angiogenic factor, known as VEGFR-2, is overexpressed in a plethora of cancers, including PTC, where it aids in tumor growth. Suppression of tumor angiogenesis by targeting VEGFR-2 makes this approach highly promising (Dhar et al., 2022). FDA-approved inhibitors of VEGFR-2 do exist, but their associated side effects of other such treatment options have become unsatisfactory (Claesson‐Welsh and Welsh, 2013). Natural products contain diverse structures and biologically active compounds that can be selectively applied with reduced side effects. Thus, we aim to design putative inhibitors of VEGFR2 using virtual docking, MD simulations, and BFE approaches. This study explored VEGFR-2 inhibitors from the African natural product database to develop effective treatments for PTC. The overall workflow of this study is shown in the Figure 1.

### Screening of AfroDb natural product database against VEGFR2 protein

**FIGURE 2:**
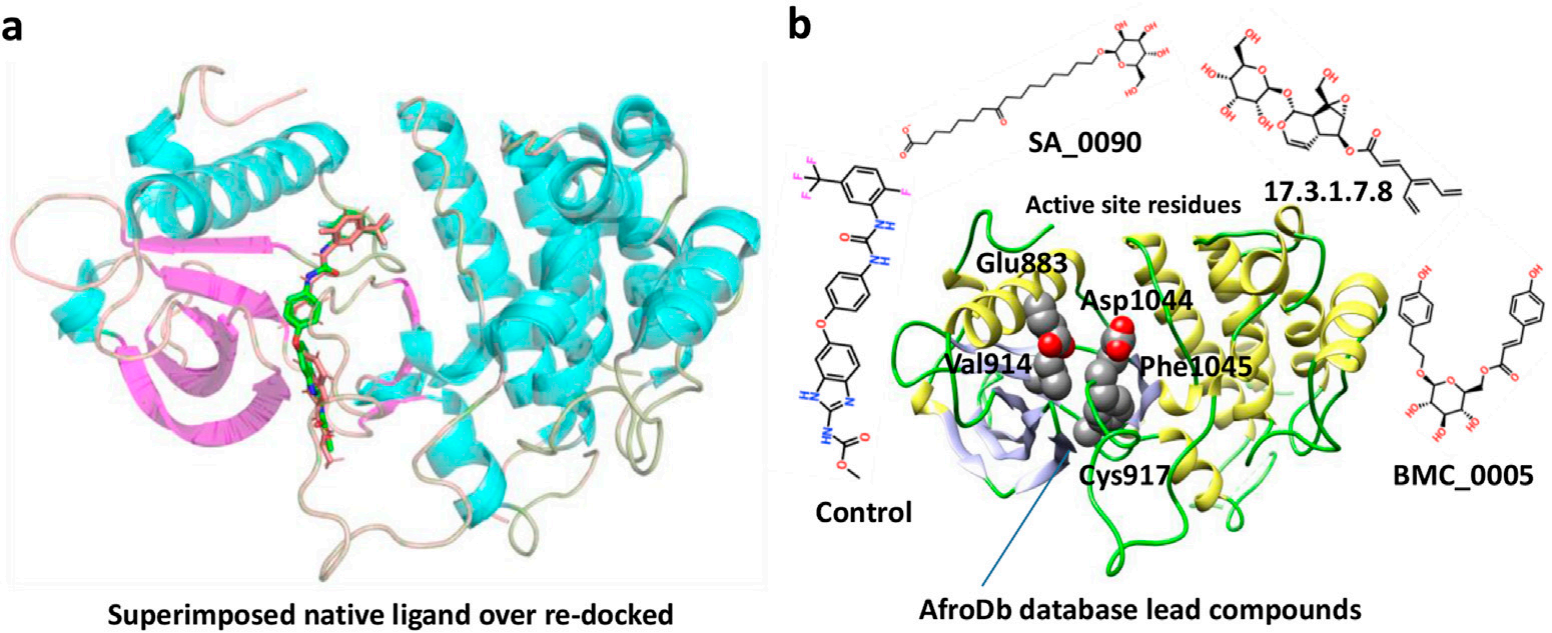
Representation of VEGFR2 active site and validation of docking accuracy. **(a)** Represents the superimposition of native ligand (Green) over re-docked ligand (orange). **(b)** Represents the active sites of VEGFR2 and the shortlisted compounds.

The Virtual drug screening (VDS) is an innovative approach in current drug discovery because it utilizes natural phytocompounds as a prospective source of therapeutics in an economical and efficient manner (Giordano et al., 2022). The unique structural and bioactive characteristics of phytocompounds allow them to be quickly screened for target specific interactions through virtual drug screening techniques. This enables the rapid discovery of novel drug candidates that possess good pharmacokinetics and low toxicity, and decreases the need for time extensive and costly experimental screening. Moreover, VDS offers the possibility of repurposing phytochemicals for several diseases and supports the design of herbal remedies by scientifically confirming their medicinal value. VDS combines herbal products with technology which increases the ability to discover new drugs for cancer, infectious diseases, and other complicated health issues (Oliveira et al., 2023) (Lin et al., 2020). In this study, we conducted virtual screening of the AfroDB database to identify potential inhibitors targeting the active site (Glu883, Val914, Cys917, Asp1044, and Phe1045) of VEGFR2 protein as shown in the Figure 2b. AfroDB is a valuable repository of bioactive compounds derived from African medicinal plants. To refine our selection, we applied Lipinski’s Rule of Five, a widely accepted criterion for assessing drug-like properties, eliminating non-compliant molecules before proceeding with screening (Suleman et al., 2024a). Our computational approach utilized AutoDock Vina for molecular docking against VEGFR2. The initial dataset comprised 954 compounds, which was reduced to 743 after filtering for drug-likeness. These compounds underwent a multi-step screening process. In the primary docking stage, binding affinities ranged from −8.839 to 5.27 kcal/mol, leading us to shortlist 54 compounds with docking scores between −7.601 and −8.839 kcal/mol. These top candidates were then subjected to induced-fit docking, refining the selection further with binding affinities between −7.37 and −9.04 kcal/mol. On the basis of high docking score only three compounds such as 8-oxo-16-[(2R, 3S, 4S, 5S, 6R)-3,4,5-trihydroxy-6-(hydroxymethyl) tetrahydropyran-2-yl]oxy-hexadecanoic, [(1aR, 1bR, 2R, 5aR, 6S, 6aR)-1a-(hydroxymethyl)-2-(2S, 3R, 4R, 5S, 6R)-3,4,5-trihydroxy-6-(hydroxymethyl)te and (2S, 3R, 4R, 5S, 6S)-3,4,5-trihydroxy-6-[2-(4-hydroxyphenyl) ethoxy]tetrahydropyran-2-yl]methyl with a docking scores of −9.04 kcal/mol, −8.96 kcal/mol, and −8.33 kcal/mol were further processed for the bonding network and stability evaluation. The selected compounds with docking scores and binding residues are shown in the Table 1. Furthermore, to validate the docking protocol, a re-docking experiment was conducted using the reference ligand of VEGFR2. This process involved reintroducing the native ligand into the active site, successfully replicating its original binding conformation. The analysis confirmed the reliability of the docking approach by demonstrating a close match with the binding pattern observed in the downloaded ligand structure. The superimposed native ligand on over re-docked is shown in the Figure 2a.

**TABLE 1:** List of lead compounds from African natural compounds and TCM database.

Database ID AfroDb ID	Compounds name	2D structure	Docking score	Interaction Type	Residues
Control	methyl (5-{4-[({[2-fluoro-5-(trifluoromethyl)phenyl]amino}carbonyl)amino]phenoxy}-1h-benzimidazol-2-yl)carbamate	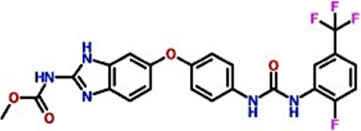	−8.39 kcal/mol	HB	Glu883
				HB	Asp1044
SA_0090	8-oxo-16-[(2R, 3S, 4S, 5S, 6R)-3,4,5-trihydroxy-6-(hydroxymethyl) tetrahydropyran-2-yl]oxy-hexadecanoic	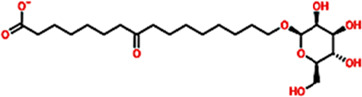	−9.04 kcal/mol	HB	Cys917
				HB	Asn921
				HB	His1024
				HB	Asp1044
				HB	Arg1049
17.3.1.7.8	[(1aR, 1bR, 2R, 5aR, 6S, 6aR)-1a-(hydroxymethyl)-2-[(2S, 3R, 4R, 5S, 6R)-3, 4, 5-trihydroxy-6-(hydroxymethyl)te	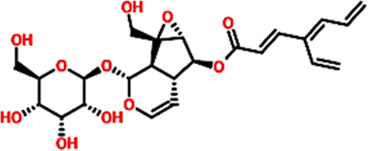	−8.96 kcal/mol	HB	Glu883
				HB	Lys866
				HB	Val912
				HB	Asp1044
BMC_0005	[(2S,3R, 4R, 5S, 6S)-3,4,5-trihydroxy-6-[2-(4-hydroxyphenyl) ethoxy]tetrahydropyran-2-yl]methyl	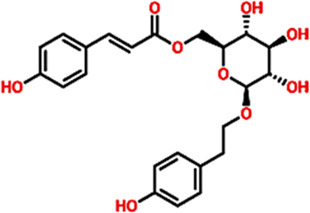	−8.33 kcal/mol	HB	Glu883
				HB	Lys866
				HB	Ile1023
				HB	Asp1044
				HB	Phe1045

### Bonding network analysis of lead compounds-VEGFR2 complexes

**FIGURE 3:**
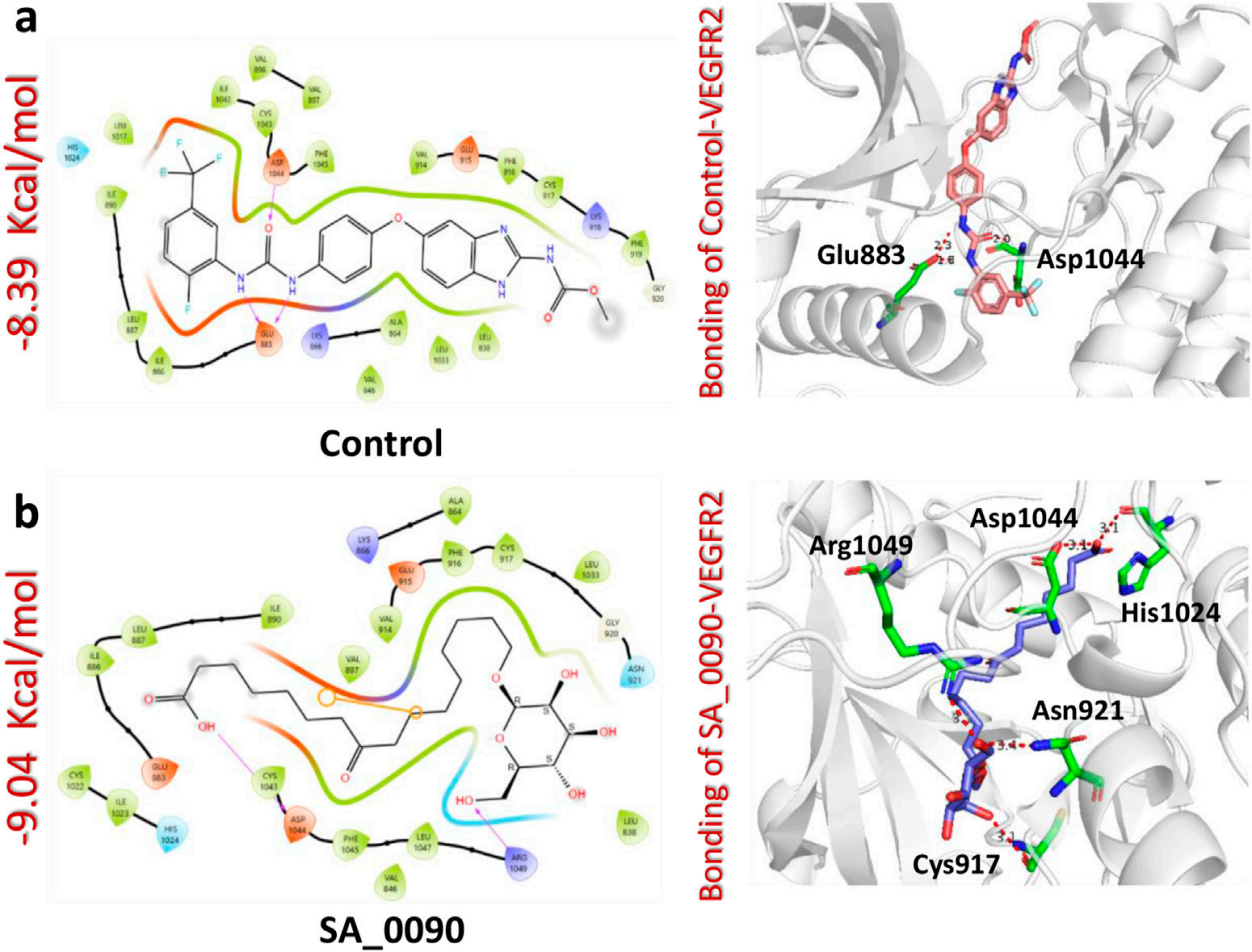
Docking analysis of control and SA_0090-VEGFR2 complexes. **(a)** Represents the interaction (2D and 3D) of control and VEGFR2 protein **(b)** Represents the interaction (2D and 3D) of SA_0090 and VEGFR2 protein.

Binding network analysis of protein-drug complexes is essential for understanding the molecular interactions that drive drug efficacy, specificity, and stability. By examining non-covalent interactions such as hydrogen bonds, hydrophobic contacts, electrostatic forces, and van der Waals interactions, researchers can identify critical residues involved in binding and optimize drug candidates for improved affinity and selectivity (Klebe, 2025). The binding analysis of the control drug (benzimidazole-urea inhibitor) demonstrated a docking score of −8.39 kcal/mol. This compound formed two hydrogen bonds with Glu883 and Asp1044 residues within the active site (Figure 3a). In contrast, the interaction analysis of the shortlisted compound SA_0090 revealed a stronger binding affinity with a docking score of −9.04 kcal/mol. SA_0090 established five hydrogen bonds with key amino acid residues, including Cys917, Asn921, His1024, Asp1044, and Arg1049 (Figure 3b). Notably, two of these residues, Cys917 and Asp1044, have previously been identified as critical for ligand binding (Elkaeed et al., 2022), suggesting that SA_0090 interacts with essential regions of the active site. Additionally, the increased number of hydrogen bonds indicates more extensive molecular interactions, which may contribute to greater binding stability and specificity. In conclusion, the higher docking score and increased number of hydrogen bond interactions suggest that SA_0090 exhibits stronger and more specific binding to the target protein compared to the control drug.

**FIGURE 4:**
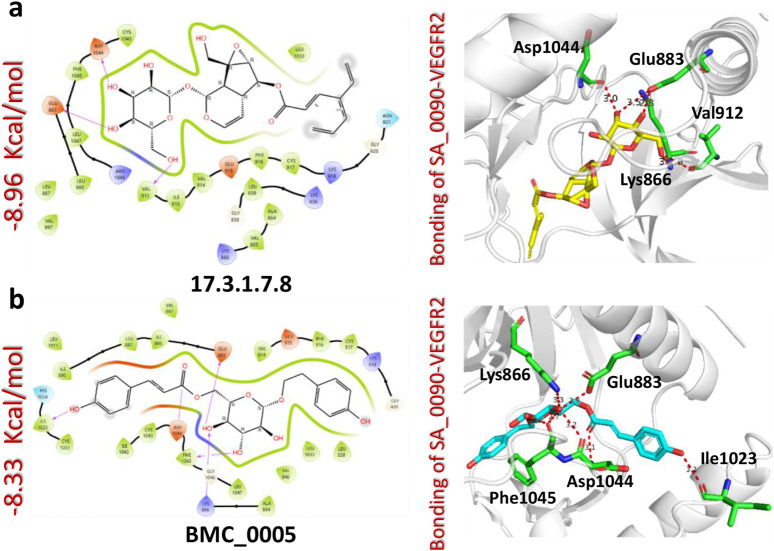
Docking analysis of 17.3.1.7.8 and BMC_0005-VEGFR2 complexes. **(a)** Represents the interaction (2D and 3D) of 17.3.1.7.8 and VEGFR2 protein **(b)** Represents the interaction (2D and 3D) of BMC_0005 and VEGFR2 protein.

The docking analysis of the 17.3.1.7.8-VEGFR2 complex revealed a docking score of −8.96 kcal/mol, indicating a stronger binding affinity compared to the control drug (−8.39 kcal/mol). This compound formed four hydrogen bonds with key amino acid residues within the active site of the VEGFR2 protein, specifically Glu883, Lys866, Val912, and Asp1044. Notably, three of these residues Glu883, Val912, and Asp1044 were previously identified as critical drug targets, reinforcing the biological relevance of these interactions. The higher docking score and the presence of key hydrogen bonds suggest that the 17.3.1.7.8 compound exhibits enhanced binding affinity and specificity relative to the control drug (Figure 4a). In comparison, the BMC_0005-VEGFR2 complex showed a slightly lower docking score of −8.33 kcal/mol, forming five hydrogen bonds with essential residues, including Glu883, Lys866, Ile1023, Asp1044, and Phe1045 (Figure 4b). The interaction of BMC_0005 with these key amino acids suggests effective binding; however, the slightly lower docking score indicates a reduced binding affinity compared to the 17.3.1.7.8 compound. In conclusion, the compounds SA_0090 and 17.3.1.7.8 demonstrated stronger binding affinity and increased molecular interactions with VEGFR2 compared to the control drug. These findings suggest their potential as more effective VEGFR2 inhibitors.

### Lipinski’s rule of five evaluation for the selected compounds

Lipinski’s Rule of Five (Ro5) is a crucial guideline in drug discovery used to evaluate the drug-likeness and oral bioavailability of compounds (Nhlapho et al., 2024). It helps predict whether a molecule can be efficiently absorbed by the human body based on key physicochemical properties: molecular weight (≤500 Da), lipophilicity (LogP ≤5), hydrogen bond donors (≤5), and hydrogen bond acceptors (≤10). By applying Ro5 early in drug development, researchers can optimize compounds for better pharmacokinetics, reduce failures in later clinical trials, and enhance the efficiency of medicinal chemistry efforts (Nhlapho et al., 2024). Therefore, to check the physiochemical properties of our selected compounds we performed the Lipinski’s rule of five evaluation for each compound. As shown in the Table 2 all compounds except the control meet the molecular weight criterion (≤500 Da), with the control slightly exceeding it (503.412 Da). “SA_0090″and “BMC_0005″fully comply with Ro5, having zero violations, making them strong drug candidates. “17.3.1.7.8″has one violation due to an excess of HBAs (11 instead of ≤10), which may impact solubility. The control compound has two violations, exceeding the limits for both molecular weight and Log P (6.3354), making it more lipophilic and potentially less soluble. The bioavailability scores range from 0.11 to 0.55, with “SA_0090″showing the lowest (0.11) due to its high polarity, while “17.3.1.7.8″and “BMC_0005″have 0.55, suggesting better oral absorption.

**TABLE 2:** Lipinski’s rule five analysis for all selected top hits.

Drugs ID	Molecular Weight	Hydrogen Acceptors	Hydrogen Donors	Consensus log P	Lipinski’s rule		Bioavailability
					Results	Violation	
Control	503.412	5	4	6.3354	Yes	2	0.55
SA_0090	447.545	9	4	0.1932	Yes	0	0.11
17.3.1.7.8	494.493	11	5	−1.1848	Yes	1	0.55
BMC_0005	446.452	9	5	0.7211	Yes	0	0.55

### ADMET properties (absorption, distribution, metabolism, excretion, toxicity) analysis of selected compounds

ADMET (Absorption, Distribution, Metabolism, Excretion, and Toxicity) evaluation is crucial in drug discovery as it ensures the selection of compounds with optimal pharmacokinetic and safety profiles. It helps enhance bioavailability, predict toxicity risks, and improve drug distribution, reducing failures in clinical trials. Early ADMET screening aids in eliminating unsuitable candidates, optimizing molecular structures, and ensuring compliance with regulatory guidelines, ultimately saving time and costs. By integrating computational and experimental ADMET assessments, researchers can develop safer and more effective drugs, increasing their chances of success in therapeutic applications. As shown in the Table 3 the ADMET evaluation of four compounds (control, SA_0090, 17.3.1.7.8, and BMC_0005) reveals distinct pharmacokinetic and toxicological profiles. In terms of absorption, all compounds have moderate water solubility (Log S values between −2.886 and −3.51), but their Caco-2 permeability varies significantly, with SA_0090 showing the lowest (−0.278) and 17.3.1.7.8 the highest (0.368). Caco-2 cells, derived from human colorectal adenocarcinoma, serve as a standard model for assessing the permeability of substances across the intestinal epithelium (Kus et al., 2023). Furthermore, the control exhibits the highest intestinal absorption (88.506%), while SA_0090 has the lowest (25.89%), with 17.3.1.7.8 and BMC_0005 showing moderate absorption (46.488% and 49.305%, respectively). In terms of distribution, none of the compounds cross the blood-brain barrier, and all localize in mitochondria. Volume of distribution (VDss) suggests SA_0090 has limited tissue distribution (−1.26), whereas BMC_0005 (0.554) distributes more widely. The volume of distribution (VD) measures how a drug disperses between plasma and tissues. If the VD value is lower than −0.15, the drug is more likely to remain in plasma rather than being distributed into tissues. Conversely, a VD value exceeding 0.45 indicates a broader distribution across tissues. Metabolically, none of the compounds act as CYP2D6 or CYP3A4 substrates, but the control inhibits CYP1A2 and CYP2C19, potentially leading to drug-drug interactions. Excretion data show SA_0090 has the highest clearance (1.915), meaning it is rapidly eliminated, whereas BMC_0005 has the lowest clearance (0.127), suggesting longer retention. None of the compounds are substrates for renal OCT2 transporters. Toxicity assessment indicates that all compounds are free from AMES toxicity, skin sensitization, carcinogenicity, and respiratory toxicity; however, the control is hepatotoxic, whereas the other three compounds are not. Overall, our selected compounds exhibit favorable pharmacokinetics properties making them promising alternatives to the control drug.

**TABLE 3:** Evaluation of ADMET properties of selected compounds.

Properties	Control	SA_0090	17.3.1.7.8	BMC_0005
Absorption				
Water solubility Log S	−2.944	−2.886	−3.51	−2.912
Caco-2 permeability × 10–6	0.035	−0.278	0.368	0.063
Human Intestinal absorption (%)	88.506	25.89	46.488	49.305
Distribution				
VDss (human)	0.214	−1.26	−0.31	0.554
BBB permeability	No	No	No	No
CNS permeability	−3.056	−4.07	−3.816	−3.62
Subcellular localization	Mitochondria	Mitochondria	Mitochondria	Mitochondria
Metabolism				
CYP2D6 substrate	No	No	No	No
CYP3A4 substrate	No	No	No	No
CYP1A2 inhibitor	Yes	No	No	No
CYP2C19 inhibitor	Yes	No	No	No
CYP3A4 inhibitor	No	No	No	No
Excretion				
Total Clearance	0.213	1.915	1.357	0.127
Renal OCT2 substrate	No	No	No	No
Toxicity				
AMES toxicity	No	No	No	No
Skin sensitization	No	No	No	No
Hepatotoxicity	Yes	No	No	No
Carcinogens	No	No	No	No
Respiratory diseases	Safe	Safe	Safe	Safe

### Post-simulation stability analysis of shortlisted compounds-VEGFR2 complexes

**FIGURE 5:**
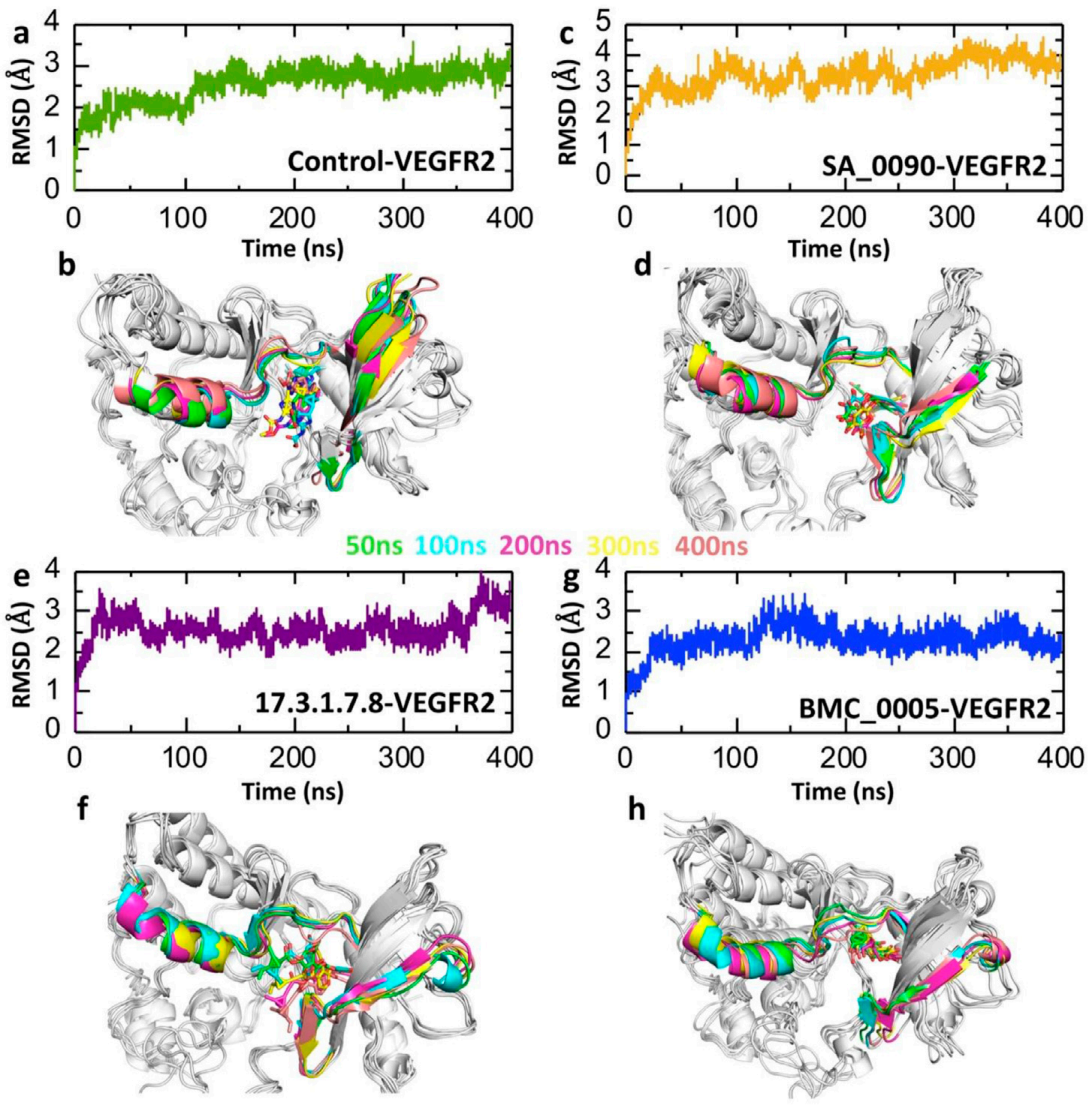
RMSD trajectories analysis for the stability of shortlisted compounds-VEGFR2 complexes. **(a,b)** Represents the dynamic stability of control drug-VEGFR2 complex. **(c,d)** Represents the dynamic stability of SA_0090-VEGFR2 complex. **(e,f)** Represents the dynamic stability of 17.3.1.7.8-VEGFR2 complex. **(g,h)** Represents the dynamic stability of BMC_0005-VEGFR2 complex.

Post-simulation RMSD (Root Mean Square Deviation) analysis is crucial for evaluating the stability, conformational changes, and binding behavior of drug-protein interactions during molecular dynamics simulations. It helps determine whether the complex reaches equilibrium, with stable RMSD values indicating structural stability and significant fluctuations suggesting conformational shifts or ligand dissociation (Hassan et al., 2024). Analyzing the RMSD of the ligand within the binding pocket reveals how well the drug remains bound, while comparing RMSD across multiple drug candidates helps identify the most stable and effective compounds. Additionally, RMSD analysis can detect binding site rearrangements, ensure the reproducibility of simulations, and validate computational findings against experimental data, making it an essential tool in rational drug design and optimization (Pieroni et al., 2023). Therefore, to check the dynamic stability of shortlisted compounds-VEGFR2 complexes we calculated the RMSD over 400 ns simulation. As shown in the Figure 5 the post-simulation RMSD (Root Mean Square Deviation) analysis of VEGFR2 complexes with various drug candidates provides insights into the structural stability and dynamic behavior of each system over a 400 ns molecular dynamics (MD) simulation. The control VEGFR2 complex maintains a relatively stable RMSD between 2 and 3 Å over the entire 400 ns simulation (Figure 5a) which also validated by the superimposition of post-simulation retrieved structures (Figure 5b). The SA_0090-VEGFR2 complex exhibits minor variation among the analyzed complexes, with values fluctuating between 3 and 4 Å. This increased RMSD suggests that the complex undergoes substantial structural rearrangements throughout the simulation. The early stages of the simulation show a gradual increase in RMSD, indicating an initial conformational adjustment and accommodation for the ligand in the active site pocket (Figure 5c). However, the structural alignment of snapshots extracted from the simulation trajectories at 50 ns, 100 ns, 200 ns, 300 na and 400 ns demonstrated that the ligand remained stably positioned within the binding pocket throughout the simulation (Figure 5d). In contrast, the 17.3.1.7.8-VEGFR2 complex shows consistent RMSD values (∼3 Å) with minimal fluctuations, indicating stable binding and limited structural perturbation as compared to the control complex (Figure 5e). Additionally, the post-simulation retrieved structure further validated the stable binding of compounds in the active site throughout the simulation (Figure 5f). Similarly, the BMC_0005-VEGFR2 complex also demonstrates a relatively stable RMSD profile, maintaining values between 2 and 3 Å (Figure 5g). This stability suggests strong and consistent ligand binding, which preserves the protein’s structural integrity. The structural alignment in Figure 5h confirms these findings, showing limited conformational changes across the simulation timeframe. The ligand appears to remain anchored within the binding pocket. Overall, the RMSD data suggest that the 17.3.1.7.8 and BMC_0005 compounds form more stable VEGFR2 complexes, while SA_0090 leads to structural deviations.

### Post-simulation fluctuation analysis of shortlisted compounds-VEGFR2 complexes at residues level

**FIGURE 6:**
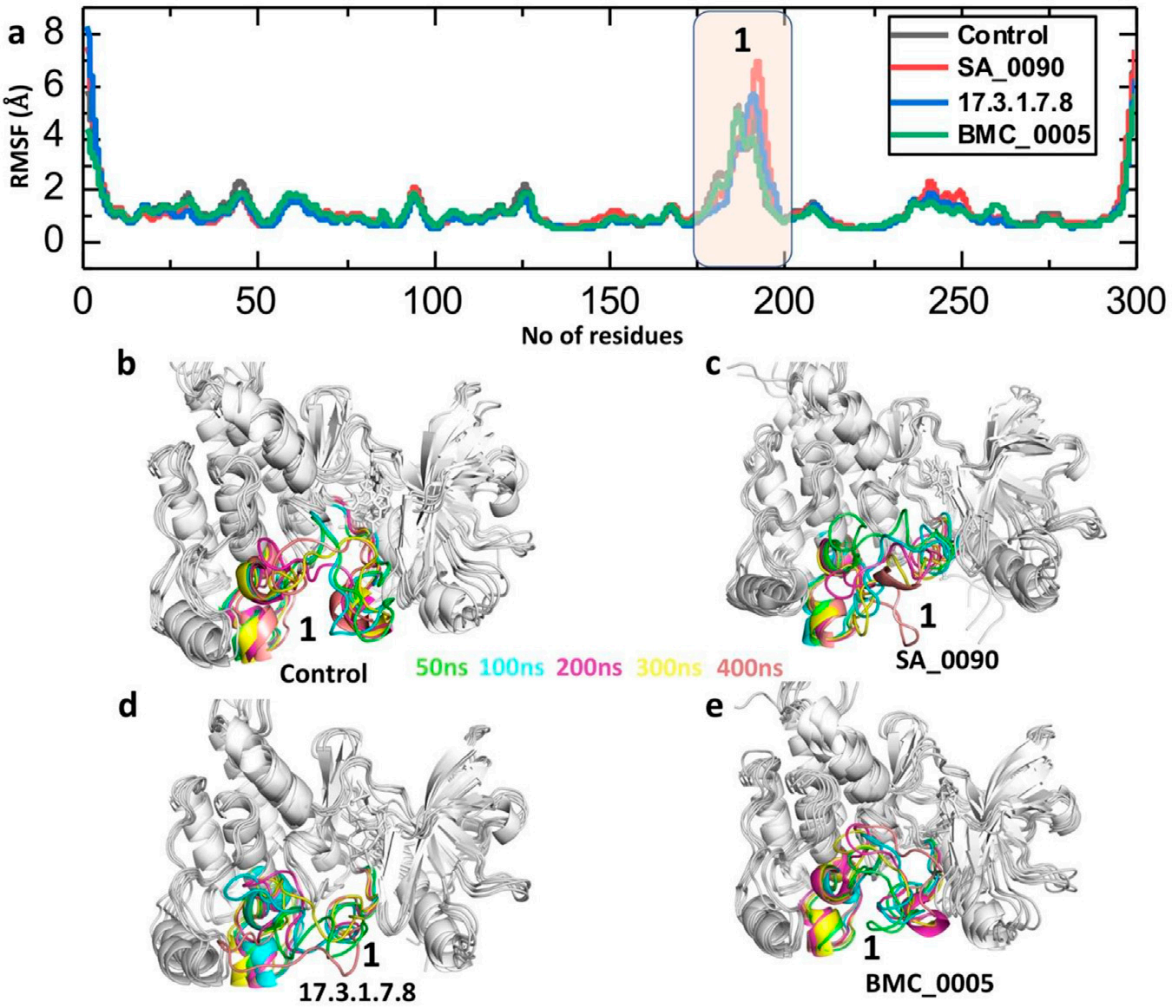
Residual fluctuation analysis of shortlisted compounds-VEGFR2 complexes by processing the RMSF trajectories. **(a)** Showing the fluctuation of each residues in control and shortlisted compounds-VEGFR2 complexes. **(b–e)** Showing the superimposition of 3D structures retrieved at different time point of simulation.

RMSF (Root Mean Square Fluctuation) analysis is crucial in drug-protein interaction studies as it provides insights into the flexibility and dynamic behavior of protein residues during molecular dynamics simulations. It helps identify flexible and rigid regions, assess the stability of the drug-binding site, and reveal conformational changes induced by ligand binding. Reduced fluctuations in the binding site indicate stable drug binding, while increased fluctuations suggest weak or unstable interactions. This analysis is valuable for optimizing drug design by targeting dynamic hotspots and comparing the effects of different drug candidates on protein stability (Yang and Kar, 2024) (Abdullahi et al., 2024). Consequently, we calculated the RMSF to evaluate the flexibility of compounds-VEGFR2 complexes at residues level. As shown in the Figure 6 the RMSF analysis of the VEGFR2-ligand complexes highlights the flexibility of individual residues throughout the 400 ns molecular dynamics simulation. The RMSF values for the control, SA_0090, 17.3.1.7.8, and BMC_0005 complexes generally remain low (<2 Å) across most residues, indicating overall structural stability. However, a significant peak is observed around residue ∼190–210, suggesting increased flexibility in this loop region across all systems. This region is associated with the ligand-binding pocket, where interactions with the drug candidates induce structural adjustments. Among the complexes, the SA_0090 exhibits slightly higher fluctuations in this region compared to others (Figure 6a). The superimposition of retrieved post-simulation 3D structures revealed that the fluctuation was primarily localized to the loop regions. This indicates that the intrinsic flexibility of these loops likely drives the observed variations, potentially influencing the dynamic behavior of drug-protein interaction (Figures 6b–e). RMSF results further validated the RMSD data showing the stable binding of shortlisted compounds in the active site of VEGFR2 protein.

### Structural compactness analysis of shortlisted compounds-VEGFR2 complexes

**FIGURE 7:**
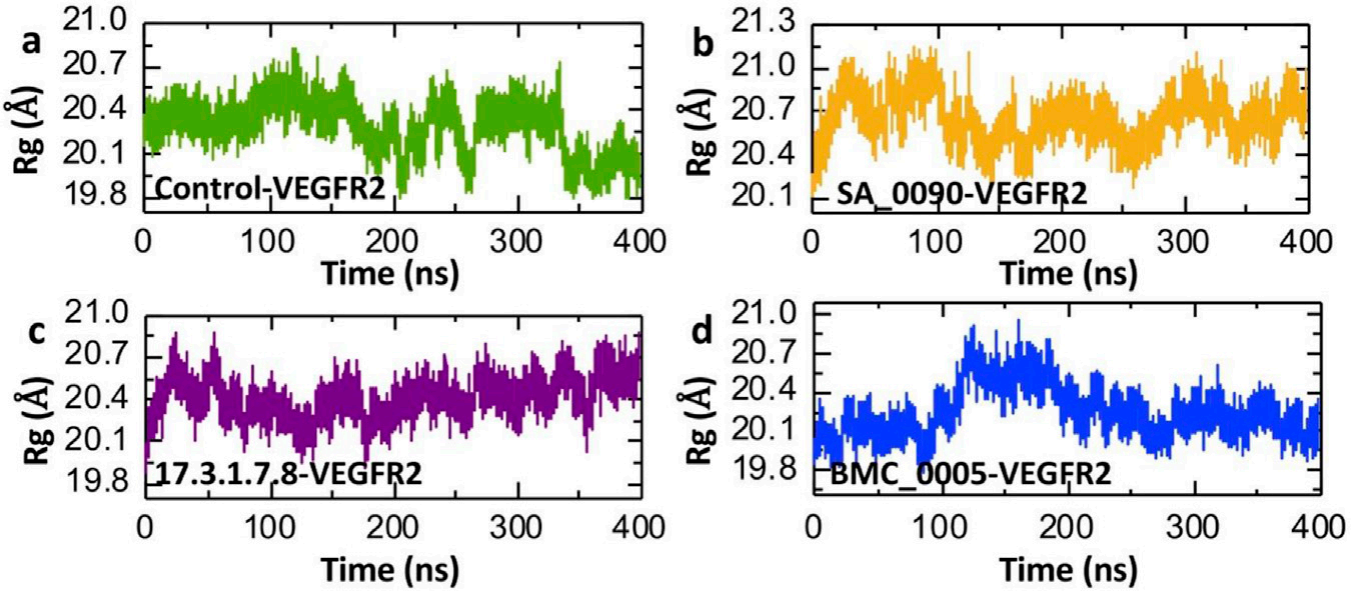
Compactness analysis of shortlisted compounds-VEGFR2 complexes by calculating Rg. **(a)** Illustrating the structural compactness of control-VEGFR2 complex. **(b)** Illustrating the structural compactness of SA_0090-VEGFR2 complex. **(c)** Illustrating the structural compactness of 17.3.1.7.8-VEGFR2 complex. **(d)** Illustrating the structural compactness of BMC_0005-VEGFR2 complex.

The radius of gyration (Rg) calculation is crucial for understanding drug-protein interactions as it provides insights into the conformational variations of the protein-ligand complex. Rg measures the distribution of atomic mass around the center of mass, indicating the compactness or flexibility of the system. A significant change in Rg values during molecular dynamics simulations suggests structural rearrangements, which can reflect the binding strength and stability of the drug-protein complex (Suleman et al., 2024a) (Khan et al., 2022). The Radius of Gyration (Rg) plots depict the compactness and structural stability of the VEGFR2 protein in complex with shortlisted compounds over a 400 ns molecular dynamics simulation (Figure 7). In the control-VEGFR2 system, the Rg fluctuates between 19.8 Å and 20.7 Å, indicating a relatively stable and compact structure throughout the simulation (Figure 7a). The SA_0090-VEGFR2 complex exhibits a slightly higher Rg range (20.1 Å to 21.0 Å) with increased fluctuations, suggesting a more flexible and less compact structure (Figure 7b). The 17.3.1.7.8-VEGFR2 complex maintains a relatively stable Rg (19.8 Å to 20.7 Å) with minimal variation, implying that this ligand stabilizes the VEGFR2 structure effectively (Figure 7c). Similarly, the BMC_0005-VEGFR2 complex shows slight fluctuations initially but stabilizes around 20.1 Å after 200 ns, indicating that this ligand maintains the structural integrity of VEGFR2 over time (Figure 7d). Overall, the 17.3.1.7.8 and BMC_0005 provide greater stability and compactness, suggesting stronger and more consistent interactions with the VEGFR2 protein as compared to the control and SA_0090.

### Post-simulation hydrogen bonds analysis of shortlisted compounds-VEGFR2 complexes

**FIGURE 8:**
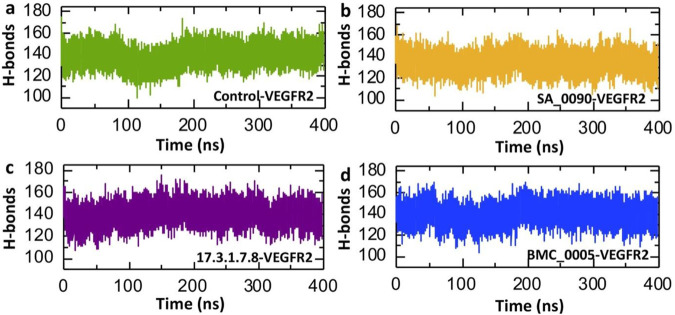
Calculation of average hydrogen bonds in shortlisted compounds-VEGFR2 complexes. **(a)** Illustrates the number of hydrogen bonds in control-VEGFR2 complex. **(b)** Illustrates the number of hydrogen bonds in SA_0090-VEGFR2 complex. **(c)** Illustrates the number of hydrogen bonds in 17.3.1.7.8-VEGFR2 complex. **(d)** Illustrates the number of hydrogen bonds in BMC_0005-VEGFR2 complex.

Post-simulation hydrogen bond analysis is crucial for understanding drug-protein interactions as it provides insights into the stability, specificity, and binding affinity of the complex under dynamic conditions. It helps assess the persistence of key hydrogen bonds, which indicates interaction stability and supports binding affinity estimation. This analysis also validates molecular docking predictions by revealing whether interactions remain stable during molecular dynamics (MD) simulations (Suleman et al., 2021). Consequently, to check the binding stability of the shortlisted compounds and VEGFR2 protein we calculated the average post-simulation hydrogen bonds. The Figure 8 presents the post-simulation analysis of hydrogen bonds (H-bonds) over 400 ns for VEGFR2 in complex with different drug candidates and a control. Figure 8a represents the control-VEGFR2 complex, showing a relatively stable H-bond count with fluctuation between 80 and 120 ns. In contrast the SA_0090-VEGFR2, 17.3.1.7.8-VEGFR2 and BMC_0005-VEGFR2 complexes showed relatively similar pattern of hydrogen bonds. The average hydrogen bonds for the control, SA_0090-VEGFR2, 17.3.1.7.8-VEGFR2 and BMC_0005-VEGFR2 complexes were recorded to be 139.14, 134.57, 139.64 and 139.63 respectively (Figures 8a–d). Across all four systems, the H-bond count remains relatively stable, suggesting the stable binding of shortlisted compounds with the active site of the VEGFR2 protein.

### Solvent Accessible Surface Area analysis of shortlisted compounds-VEGFR2 complexes

**FIGURE 9:**
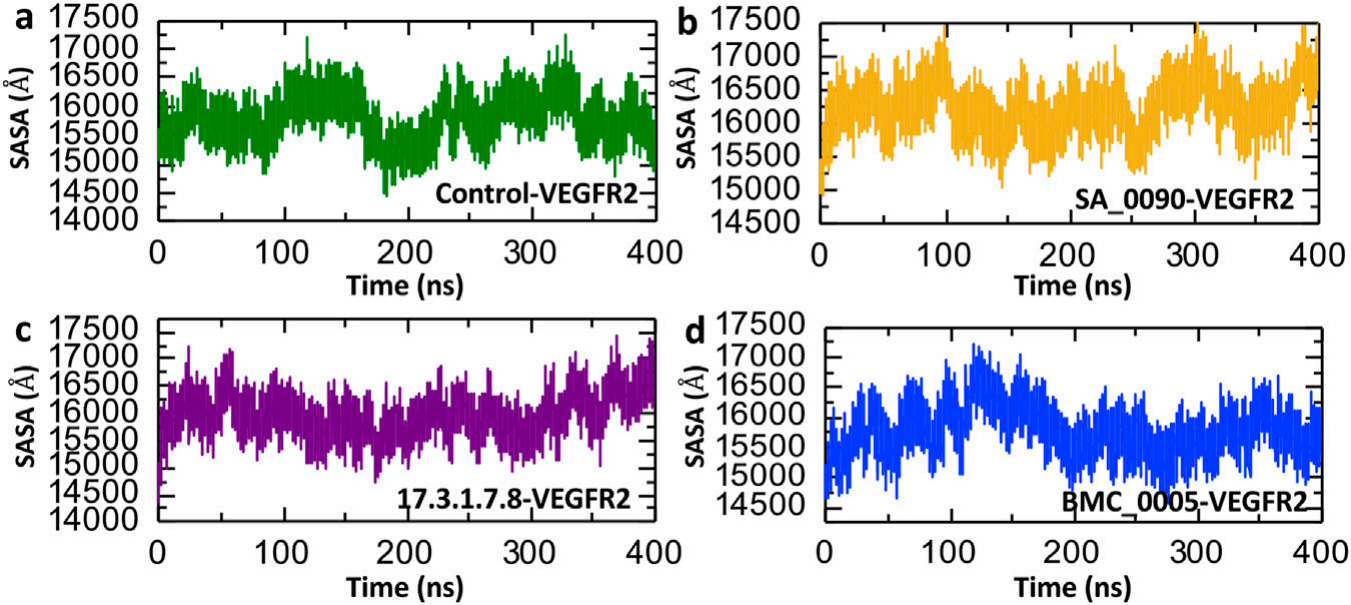
Surface area analysis of shortlisted compounds-VEGFR2 complexes. **(a)** Represents the SASA analysis of control-VEGFR2 complex. **(b)** Represents the SASA analysis of SA_0090-VEGFR2 complex. **(c)** Represents the SASA analysis of 17.3.1.7.8-VEGFR2 complex. **(d)** Represents the SASA analysis of BMC_0005-VEGFR2 complex.

Solvent Accessible Surface Area (SASA) analysis is a key tool in computational biology and structural bioinformatics. It calculates how much of a molecule’s surface is exposed to surrounding solvent, such as water. This method is widely used to study biological macromolecules like proteins, DNA, and RNA. By analyzing exposed surface areas, researchers can better understand molecular interactions, locate ligand binding sites, examine protein-protein interfaces, and predict how molecules may behave in different environments (Suleman et al., 2024b). The Figure 9 presents Solvent Accessible Surface Area (SASA) over a 400 ns molecular dynamics (MD) simulation for VEGFR2 in complex with the control and three lead compounds: SA_0090 (b), 17.3.1.7.8 (c), and BMC_0005 (d). The Control-VEGFR2 complex displays moderate fluctuations in SASA values, ranging from approximately 15,000 to 16,500 Å^2^, suggesting relatively stable but dynamic behavior (Figure 9a). In comparison, the SA_0090-VEGFR2 complex shows slightly higher and more variable SASA values, peaking near 17,500 Å^2^, indicating enhanced solvent exposure, possibly due to ligand-induced conformational flexibility (Figure 9b). The 17.3.1.7.8-VEGFR2 complex shows the most consistent SASA values, largely maintaining within the 15,000–16,500 Å^2^ range, suggesting stable ligand binding with minimal conformational disruption (Figure 9c). Conversely, the BMC_0005-VEGFR2 complex shows decreasing SASA values over time, indicating a potential compaction of the protein structure upon ligand binding (Figure 9d). Overall, the SASA trends suggest that different ligands influence VEGFR2 structural dynamics distinctively, with BMC_0005 potentially stabilizing a more compact conformation.

### Binding free energy calculation

MM/GBSA binding free energy is crucial for understanding drug-protein interactions as it provides an accurate estimation of binding affinity, helping to identify and prioritize potent drug candidates (Tuccinardi, 2021). By decomposing the total binding energy into van der Waals, electrostatics, and solvation components, it reveals the key forces driving molecular recognition and stability. This method aids in predicting the stability of drug-protein complexes during molecular dynamics simulations and guides rational drug design by highlighting areas for chemical modification to improve affinity. Additionally, MM/GBSA helps evaluate the effects of protein mutations on drug binding, offering insights into drug resistance. It complements experimental techniques by providing atomic-level details, accelerating drug discovery and optimizing lead compounds (Yau et al., 2024). Therefore, to check the binding strength of shortlisted compounds, we calculated the total binding free energy by using the MM/GBSA approach. The MM/GBSA analysis presented in Table 4 evaluates the binding free energies of the control and three shortlisted compounds (SA_0090, 17.3.1.7.8, and BMC_0005) by breaking down the energy contributions. The van der Waals (ΔEvdw) interactions remain relatively consistent across all compounds, with energies of −67.1847 ± 0.37 kcal/mol, −58.4277 ± 0.33 kcal/mol, −56.447 ± 0.45 kcal/mol and −66.7041 ± 0.32 kcal/mol for control, SA_0090, 17.3.1.7.8, and BMC_0005 respectively. However, electrostatic energy (ΔEele) is significantly more favorable in the shortlisted compounds, with SA_0090 showing the highest contribution (−109.9232 kcal/mol) compared to the control (−40.513 kcal/mol). However, the polar solvation energy (EGB) is also substantially higher for the shortlisted compounds, particularly for SA_0090 (124.6813 kcal/mol), suggesting greater desolvation penalties. The non-polar solvation energy (ESURF) remains similar across all samples, with slight variation, where SA_0090 has the largest contribution (−8.6036 kcal/mol). Despite the increased electrostatic attraction in the shortlisted compounds, the overall binding free energy (ΔG total) reveals that BMC_0005 (−61.476 kcal/mol) shows the most stable binding, as compared to the control (−60.3861 kcal/mol), while the recorded total binding free energies for 17.3.1.7.8 and SA_0090 were −52.7797 kcal/mol and −52.2732 kcal/mol, respectively. These results suggest that BMC_0005 may have the strongest binding affinity among the shortlisted compounds, while the higher solvation reduce the overall binding efficiency for 17.3.1.7.8 and SA_0090. Binding free results further validated the RMSD, RMSF and Rg data.

**TABLE 4:** List of binding free energies calculated by using the MM/GBSA.

MM/GBSA				
Parameters	Control	SA_0090	17.3.1.7.8	BMC_0005
ΔEvdw	−67.1847 ± 0.37	−58.4277 ± 0.33	−56.447 ± 0.45	−66.7041 ± 0.32
ΔEele	−40.513 ± 1.01	−109.9232 ± 1.84	−106.767 ± 3.46	−97.7591 ± 2.33
EGB	54.854 ± 0.96	124.6813 ± 1.82	117.7621 ± 3.22	110.7056 ± 1.93
ESURF	−7.5425 ± 0.02	−8.6036 ± 0.02	−7.3279 ± 0.04	−7.7184 ± 0.03
Delta G Gas	−107.6977 ± 1.05	−168.3509 ± 1.93	−163.2139 ± 3.56	−164.4632 ± 2.33
Delta G Solv	47.3116 ± 0.95	116.0777 ± 1.81	110.4342 ± 3.19	102.9872 ± 1.91
∆G total	−60.3861 ± 0.39	−52.2732 ± 0.37	−52.7797 ± 0.62	−61.476 ± 0.59

## Conclusion

Papillary thyroid carcinoma (PTC) remains a significant clinical challenge, particularly in aggressive and metastatic cases where current therapeutic options are limited. Given the critical role of VEGFR-2 in tumor angiogenesis and its overexpression in PTC, targeting this receptor presents a promising avenue for therapeutic intervention. In this study, we employed a structure-based virtual screening approach, molecular dynamics simulations, and binding free energy calculations to identify potent VEGFR-2 inhibitors from the African natural product database. Our findings revealed three lead phytocompounds SA_0090 (−9.04 kcal/mol), 17.3.1.7.8 (−8.96 kcal/mol), and BMC_0005 (−8.33 kcal/mol) that demonstrated superior binding affinities compared to the control compound (-8.39 kcal/mol). Among them, 17.3.1.7.8 and BMC_0005 exhibited remarkable stability in molecular dynamics simulations, with minimal structural fluctuations and strong binding interactions. Additionally, ADMET analysis of 17.3.1.7.8 and BMC_0005 confirmed their favorable pharmacokinetic properties, including optimal solubility, gastrointestinal absorption, and non-hepatotoxicity, making them promising drug candidates. Notably, binding free energy calculations identified BMC_0005 as the most potent inhibitor, surpassing both the control and other shortlisted compounds in overall stability and affinity. These results underscore the potential of natural product-derived VEGFR-2 inhibitors as promising therapeutic agents for PTC. The limitations of this study is the lack of experimental validation and analysis of potential off target affects. Therefore, further *in vitro* and *in vivo* studies are essential to validate their biological efficacy and safety.

## Data Availability

The original contributions presented in the study are included in the article/supplementary material, further inquiries can be directed to the corresponding authors.
